# An analysis of clinical process measures for acute healthcare delivery in Appalachia: The Roane Medical Center experience

**DOI:** 10.1186/1478-4505-4-3

**Published:** 2006-03-29

**Authors:** Karla Rae Pope, John S Hancock, Eric Scott Sills

**Affiliations:** 1 Department of Obstetrics and Gynecology, St. Matthew's University College of Medicine, Grand Cayman, British West Indies; 2 Division of Public Health Partnerships, Centers for Disease Control and Prevention, Atlanta, Georgia, USA; 3 Department of Obstetrics, Gynecology and Reproductive Research, Murphy Medical Center, Murphy, North Carolina, USA; 4 Suite D, 75 Medical Park Drive, Murphy Medical Center, Murphy, 28906, North Carolina ,USA

## Abstract

**Objective:**

To survey management of selected emergency healthcare needs in a Tennessee community hospital.

**Materials and methods:**

In this descriptive report, discharges and associated standard process measures were retrospectively studied for Roane Medical Center (RMC) in Harriman, Tennessee (pop. 6,757). Hospital data were extracted from a nationwide database of short-term acute care hospitals to measure 16 quality performance measures in myocardial infarction (MI), heart failure, and pneumonia during the 14 month interval ending March 2005. The data also permitted comparisons with state and national reference groups.

**Results:**

Of RMC patients with myocardial infarction (MI), 94% received aspirin on arrival, a figure higher than both state (85%) and national (91%) averages. Assessment of left ventricular dysfunction among heart failure patients was also higher at RMC (98%) than the state (74%) or national (79%) average. For RMC pneumonia patients, 79% received antibiotics within 4 h of admission, which compared favorably with State (76%) and national (75%) average. RMC scored higher on 13 of 16 clinical process measures (*p*<0.01, sign test analysis, >95% CI) compared to state and national averages.

**Discussion:**

Although acute health care needs are often met with limited resources in medically underserved regions, RMC performed above state and national average for most process measures assessed in this review. Our data were derived from one facility and the associated findings may not be applicable in other healthcare settings. Further studies are planned to track other parameters and specific clinical outcomes at RMC, as well as to identify specific institutional policies that facilitate attainment of target quality measures.

## Introduction

While health care quality in the U.S. has been found to be inconsistent and sometimes inadequate by several investigators [1,2], objective assessments of quality can provide useful information as quality improvement programs are developed and implemented [3]. Some researchers have observed that care for some seriously ill patients has shown surprising and unacceptable interstudy variation even when objective improvements were registered at the end of an assessment period [4-6]. As a medically underserved area, Appalachia encounters any economic downturn with special vulnerability. Hospital policymakers in this region are expected to maintain delivery of health services to their communities with resources that are often limited and insufficient. Against this background, this study examined one Appalachian hospital to determine its current performance vis-à-vis selected clinical process measurements and compared these data to comparable measurements across the state and nation.

## Materials and methods

### Study hospital

Roane Medical Center (RMC) is a 109-bed primary care hospital owned and operated by the city of Harriman, Tennessee (population 6,757). The hospital was established as Harriman City Hospital in 1939 initially opening with 50 beds. With closure of other small area hospitals over the next decades, this facility became the county's only hospital and was reincorporated as Roane Medical Center in 1996. The hospital's emergency department registered 23,879 patient encounters in 2004. For 2004, RMC's total annual operating budget was $36 M. RMC serves Roane and neighboring counties, most of which (like Roane County) have been designated medically underserved areas [7].

### Data source and statistical analysis

This descriptive study was based on national data collected by short-term acute care hospitals and rural, small, remote "critical access" hospitals and reported to the Centers for Medicare and Medicaid Services. RMC was among the reporting hospitals (*n *= 3558) that voluntarily submitted data in order to receive an incentive payment established by Section 501(b) of the Medicare Prescription Drug, Improvement and Modernization Act of 2003 (MMA). To qualify for such funds, MMA required eligible hospitals to report on an initial set of ten quality performance measures targeting patients diagnosed with acute myocardial infarction (MI), heart failure, and pneumonia and agree to make the data public. Subsequently, most hospitals agreed to participate in the Hospital Quality Alliance (HQA) – the first nationwide initiative to report on hospital performance. Beginning with the second quarter 2004 (April-June), hospitals participating in HQA could elect to submit data on an additional seven indicators of quality of care for these three diagnostic categories. Data were available for most clinical process measures for a 14 month period ending March 2005, although 2 of the parameters included in this study had a narrower measurement interval (January-March 2005).

The HQA data set provides a large quantity of information about U.S. hospitals where data on at least one stable measure (defined as discharge information derived from at least 25 patients) was reported during the study period. Our retrospective analysis was based on information extracted from this database specific to RMC, derived from administrative data and medical record documents as primary sources maintained at that facility. RMC's performance data were then referenced with state (Tennessee) and U.S. national average data for comparison using sign test methodology [8].

## Results

**Figure 1:**
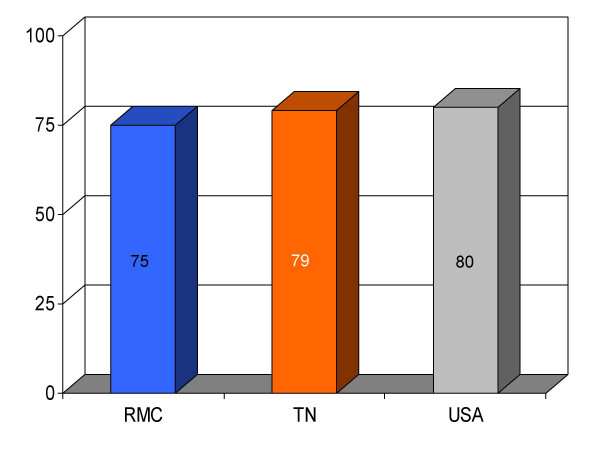
Proportion of patients with myocardial infarction (*n *= 8) given angiotensin converting enzyme inhibitor therapy for left ventricular dysfunction within 24 h of hospital admission (%) at Roane Medical Center (RMC). Mean percentages of patients receiving this treatment in Tennessee (TN) and all hospitals (USA) are also shown for comparison.

**Figure 2:**
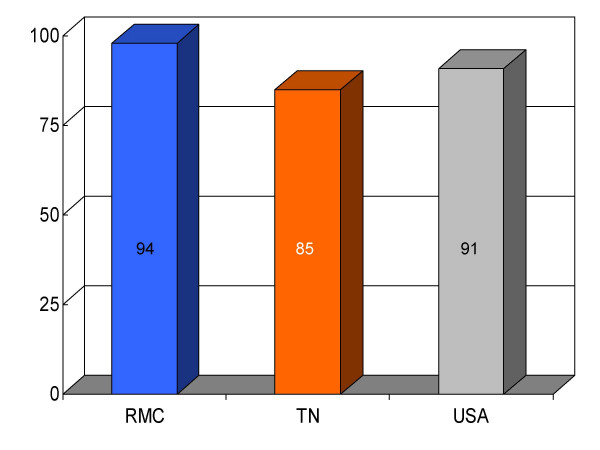
Proportion of myocardial infarction patients (*n *= 47) given aspirin upon arrival at Roane Medical Center (RMC). Mean percentages of patients receiving this treatment in Tennessee (TN) and all hospitals (USA) are also shown for comparison.

**Figure 3:**
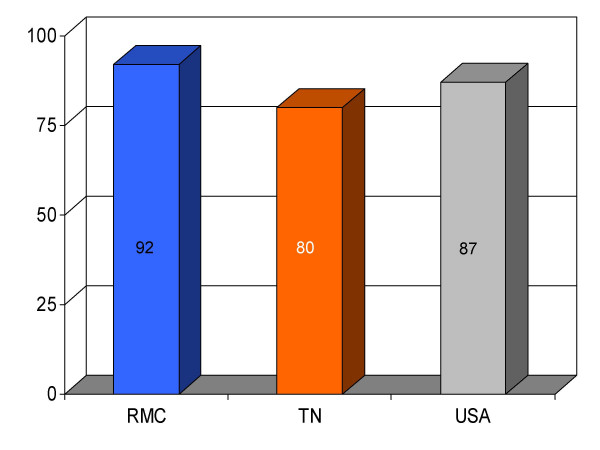
Proportion of myocardial infarction patients (*n *= 25) given aspirin at discharge from Roane Medical Center (RMC). Mean percentages of patients receiving this intervention in Tennessee (TN) and all hospitals (USA) are also shown for comparison.

**Figure 4:**
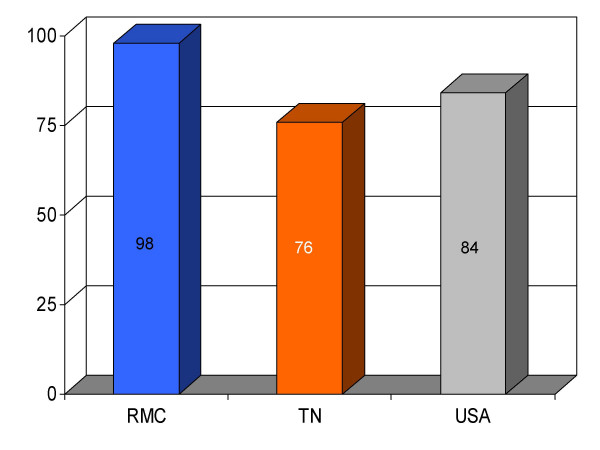
Proportion of myocardial infarction patients (*n *= 42) given beta-blocker upon arrival at Roane Medical Center (RMC). Mean percentages of patients receiving this intervention in Tennessee (TN) and all hospitals (USA) are also shown for comparison.

**Figure 5:**
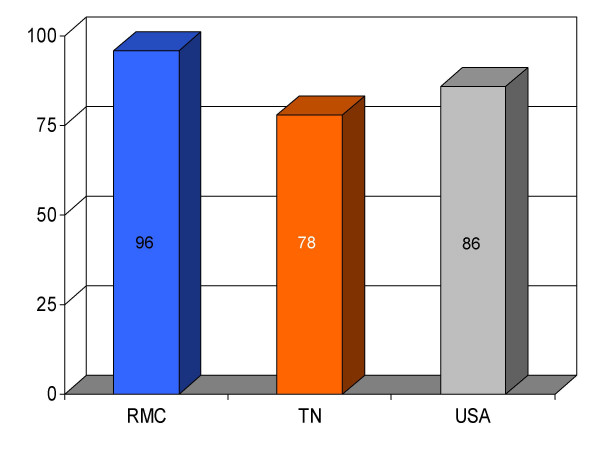
Proportion of myocardial infarction patients (*n *= 25) given beta-blocker at discharge from Roane Medical Center (RMC). Mean percentages of patients receiving this intervention in Tennessee (TN) and all hospitals (USA) are also shown for comparison.

**Figure 6:**
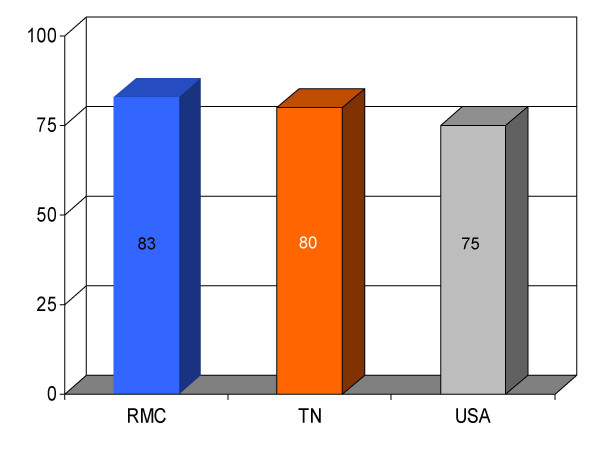
Proportion of myocardial infarction patients (*n *= 6) given smoking cessation counseling/advice before discharge from Roane Medical Center (RMC). Mean percentages of patients receiving this intervention in Tennessee (TN) and all hospitals (USA) are also shown for comparison.

**Figure 7:**
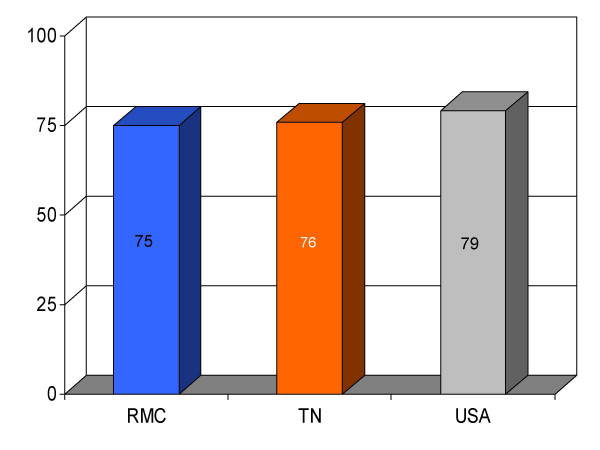
Proportion of patients with left ventricular dysfunction (*n *= 16) given an angiotensin converting enzyme inhibitor within 24 h of admission at Roane Medical Center (RMC). Mean percentages of patients receiving this intervention in Tennessee (TN) and all hospitals (USA) are also shown for comparison.

**Figure 8:**
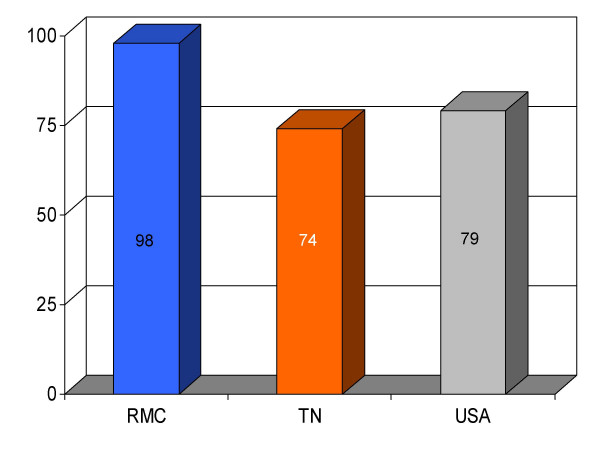
Proportion of heart failure patients (*n *= 148) receiving left ventricular function assessment at Roane Medical Center (RMC). Mean percentages of patients receiving this intervention in Tennessee (TN) and all hospitals (USA) are also shown for comparison.

**Figure 9:**
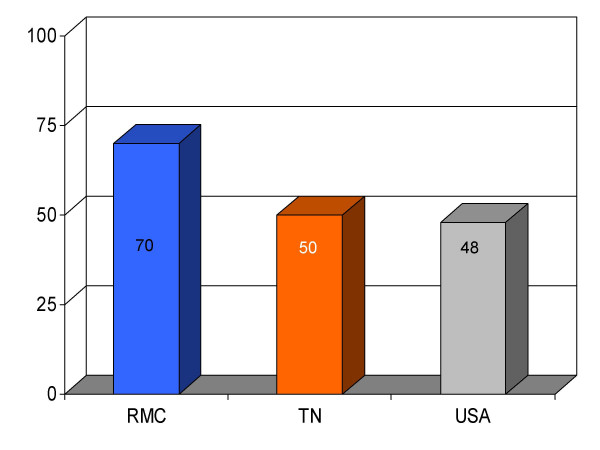
Proportion of heart failure patients (*n *= 102) receiving instructions at discharge from Roane Medical Center (RMC). Mean percentages of patients receiving discharge instructions in Tennessee (TN) and all hospitals (USA) are also shown for comparison.

**Figure 10:**
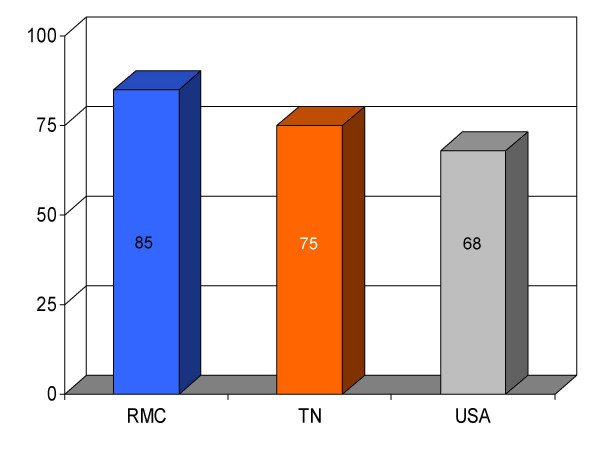
Proportion of patients with left ventricular systolic dysfunction (*n *= 26) given smoking cessation counseling/advice at Roane Medical Center (RMC). Mean percentages of patients receiving this intervention in Tennessee (TN) and all hospitals (USA) are also shown for comparison.

**Figure 11:**
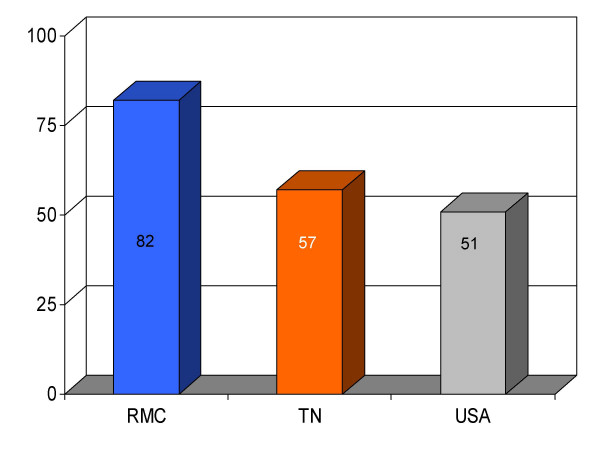
Proportion of pneumonia patients (*n *= 195) receiving pneumococcal vaccination before discharge from Roane Medical Center (RMC). Mean percentages of patients receiving this intervention in Tennessee (TN) and all hospitals (USA) are also shown for comparison.

**Figure 12:**
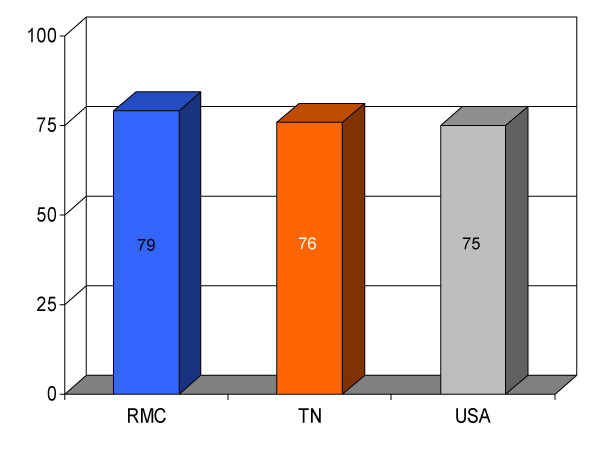
Proportion of pneumonia patients (*n *= 269) receiving intravenous antibiotics within 4 h of admission at Roane Medical Center (RMC). Mean percentages of patients receiving this treatment in Tennessee (TN) and all hospitals (USA) are also shown for comparison.

**Figure 13:**
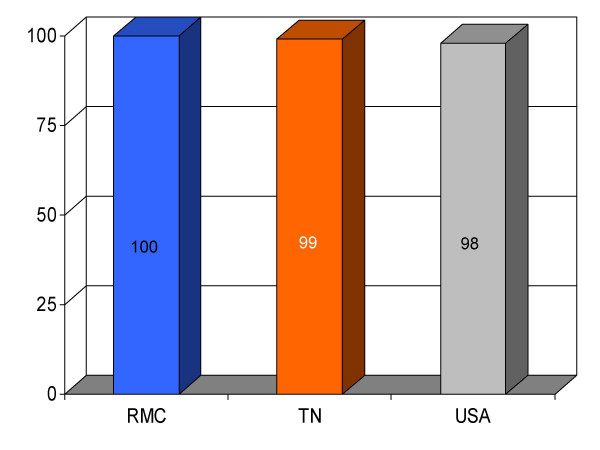
Proportion of pneumonia patients (*n *= 316) undergoing oxygenation assessment within 24 h of admission at Roane Medical Center (RMC). Mean percentages of patients having this test in Tennessee (TN) and all hospitals (USA) are also shown for comparison.

**Figure 14:**
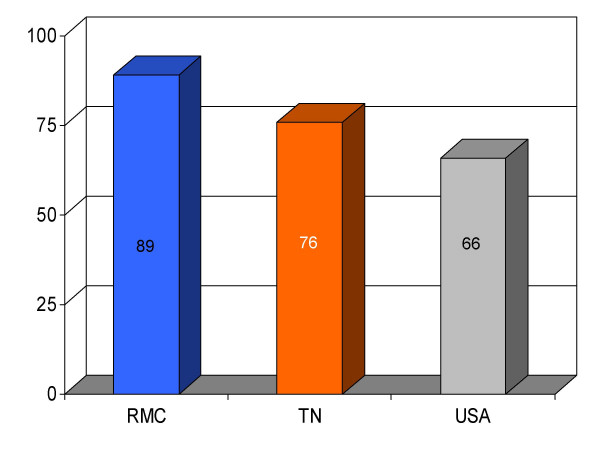
Proportion of pneumonia patients (*n *= 108) receiving smoking cessation counseling/advice from Roane Medical Center (RMC). Mean percentages of patients receiving this intervention in Tennessee (TN) and all hospitals (USA) are also shown for comparison.

**Figure 15:**
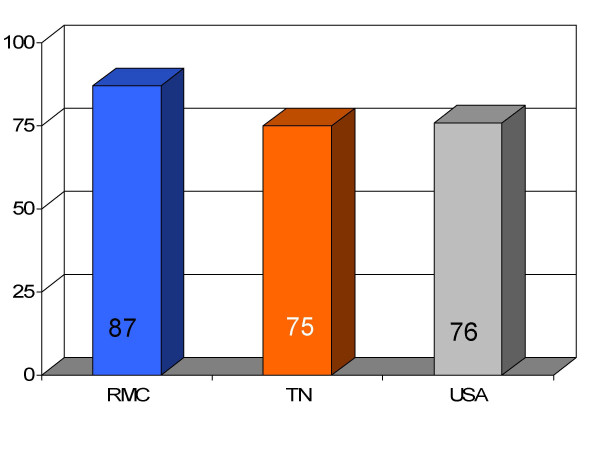
Proportion of pneumonia patients (*n *= 197) receiving most appropriate intravenous antibiotics at Roane Medical Center (RMC). Mean percentages of patients receiving this treatment in Tennessee (TN) and all hospitals (USA) are also shown for comparison.

**Figure 16:**
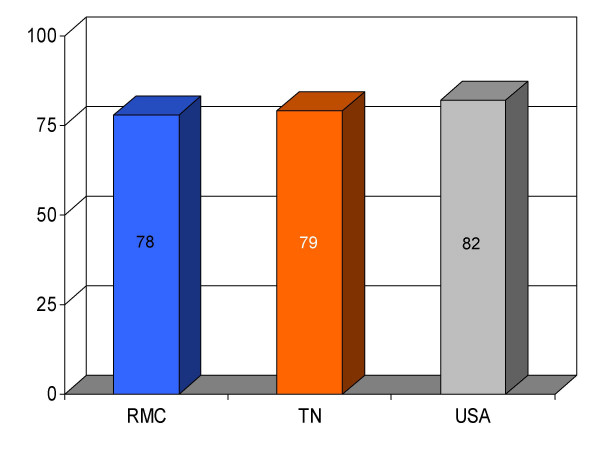
Proportion of pneumonia patients (*n *= 285) having blood cultures obtained before administration of intravenous antibiotics at Roane Medical Center (RMC). Mean percentages of patients undergoing this intervention in Tennessee (TN) and all hospitals (USA) are also shown for comparison.

We found RMC's score on a majority (13/16) of clinical parameters to be above state and U.S. national reference groups. Specifically, the study hospital's performance on utilization of aspirin on arrival and discharge for myocardial infarction was higher than the state and national average. Administration of beta-blockers for myocardial infarction at RMC was also greater compared to both Tennessee and USA average. Heart failure patients underwent assessment of left ventricular function more often at RMC compared to the state and national average, and RMC pneumonia patients were treated with intravenous antibiotics at a higher rate than state and national averages. An exception to this trend was noted in RMC's performance on angiotensin converting enzyme inhibitor use among heart attack patients. On this process measure only 78% of patients received the intervention compared to 79% and 80%, as state and national average, respectively. For uncertain reasons, the proportion of heart failure patients receiving angiotensin converting enzyme inhibitor was also nominally below standard reference groups (75% vs 76% and 79%, state and national average, respectively). Center-specific data for RMC cardiac patients given thrombolytic medication within 30 min of arrival or those receiving percutaneous coronary interventions within 120 min of arrival could not be compared to reference groups, because these services were not provided at the study hospital. RMC's performance as measured by the HQA initiative for all relevant categories is compared to state and national reference groups in figures 1, 2, 3, 4, 5, 6, 7, 8, 9, 10, 11, 12, 13, 14, 15, 16 and Table 1.

**Table 1:** Quality measure comparisons for Roane Medical Center, Tennessee state average, and USA national average for selected clinical parameters as assessed by the Hospital Quality Alliance

**Parameter**	**RMC (*n*)**	**State**	**USA**	**Comparison**
ACE inhibitor for MI^a^	75 (8)	79	80	-
ASA for MI (on arrival)	94 (47)	85	91	+
ASA for MI (at discharge)	92 (25)	80	87	+
β-blocker for MI (on arrival)	98 (42)	76	84	+
β-blocker for MI (at discharge)	96 (25)	78	86	+
Smoking cessation for MI	83 (6)	80	75	+
ACE for HF^a^	75 (16)	76	79	-
Assessment of LV function for HF	98 (148)	74	79	+
Discharge instructions for HF	70 (102)	50	48	+
Smoking cessation for HF	85 (26)	75	68	+
Vaccination for pneumonia	82 (195)	57	51	+
ABX within 4 h for pneumonia	79 (269)	76	75	+
O_2 _assessment in pneumonia	100 (316)	99	98	+
Smoking cessation for pneumonia	89 (108)	76	66	+
Most appropriate ABX for pneumonia^b^	87 (197)	75	76	+
Blood cultures for pneumonia	78 (285)	79	82	-
	
	*p *< 0.01^c^			

*Notes*: All data presented as percentages (n), and were collected from July 2004–March 2005, except as noted. *n *= total patients in criteria set (denominator) ACE = angiotensin converting enzyme inhibitor MI = myocardial infarction ASA = aspirin PCI = percutaneous coronary intervention HF = heart failure ABX = antibiotics

^a ^Data for this process measure were available for the January 2005–March 2005 interval only

^b ^Data for this process measure were available for the July 2004–March 2005 interval only

^c ^by sign test

## Discussion

Appalachia is one of several U.S. regions often identified as medically underserved [9], and its formidable healthcare needs have been described by numerous investigators [10,11]. Against this background, hospitals have confronted the challenge of diminished reimbursement, high expenses, limited staffing and other financial hardships in a variety of ways. An uncertain and difficult economic climate in the region has contributed to closure or consolidation of many small hospitals providing important primary care services to their local communities [12,13].

Several healthcare institutions in Appalachia have adapted to the changing healthcare landscape, even managing to register growth despite a hostile marketplace. One facility that has weathered this storm is Roane Medical Center (RMC), located in Harriman, Tennessee. A study of specific management strategies potentiating the survival and growth of this institution is beyond the scope of the current report. Instead, we focused on objective measurement of RMC's performance with a select group of standardized acute healthcare clinical processes as measured by the Hospital Quality Alliance (HQA). This initiative is the first effort to report data on hospital performance on a national scale [14], and permitted comparison of RMC with reference groups both within Tennessee as well as nationwide.

Research based on HQA data recently found no relationship between clinical performance on these standardized parameters and hospital size [14]. However, these investigators found a small but significant increase in performance among academic, Northeastern or Midwestern non-profit hospitals. Southern and far Western states, in contrast, tended to do less well on heart care measures [15]. Our research found RMC (a small, non-academic, not-for-profit hospital in the rural South) performed above state and national average on most of these specific clinical parameters. Indeed, the only process measures where RMC performed marginally under state and national average was among pneumonia patients receiving blood cultures, and heart failure/myocardial infarction patients receiving angiotensin converting enzyme inhibitor therapy.

Our analysis was limited by several factors. The clinical parameters covered in this report did not capture data on all hospital encounters, and represent only about 15% of all Medicare admissions [14]. It must be acknowledged that process measures are distinct from patient outcomes, and this investigation assessed only the former. Since RMC is a small facility, the number of patients presenting with certain medical conditions during the study interval was limited and likely introduced some error due to insufficient sampling. Our results describe process measures only at one institution and may not apply to different health care facilities where different policies and practices prevail. We also assumed the data reported represented independent variables, permitting robust comparisons for each process measurement. Additionally, while data collecting and public reporting of healthcare quality measures to the HQA database is an important beginning, our findings suggest that other relevant clinical benchmarks will need to be surveyed going forward. Data gathering should be expanded to include more diseases and conditions to depict a more complete picture of hospital care in rural Appalachia and nationwide. The HQA project presents valuable information for consumers, physicians, and administrators – all of whom have an interest in improving hospital care in the U.S.

## Competing interests

The author(s) declare that they have no competing interests.

## Authors' contributions

KRP, JSH and ESS contributed equally to this manuscript.
